# Effectiveness of a Voice-Based Mental Health Evaluation System for Mobile Devices: Prospective Study

**DOI:** 10.2196/16455

**Published:** 2020-07-20

**Authors:** Masakazu Higuchi, Mitsuteru Nakamura, Shuji Shinohara, Yasuhiro Omiya, Takeshi Takano, Shunji Mitsuyoshi, Shinichi Tokuno

**Affiliations:** 1 Department of Bioengineering Graduate School of Engineering The University of Tokyo Tokyo Japan; 2 Department of Pharmacology School of Medicine The University of Texas Health Science Center at San Antonio San Antonio, TX United States; 3 Research and Product Development Department PST Inc Kanagawa Japan

**Keywords:** mental health, monitoring system, stress evaluation, voice analysis

## Abstract

**Background:**

We developed a system for monitoring mental health using voice data from daily phone calls, termed Mind Monitoring System (MIMOSYS), by implementing a method for estimating mental health status from voice data.

**Objective:**

The objective of this study was to evaluate the potential of this system for detecting depressive states and monitoring stress-induced mental changes.

**Methods:**

We opened our system to the public in the form of a prospective study in which data were collected over 2 years from a large, unspecified sample of users. We used these data to analyze the relationships between the rate of continued use, the men-to-women ratio, and existing psychological tests for this system over the study duration. Moreover, we analyzed changes in mental data over time under stress from particular life events.

**Results:**

The system had a high rate of continued use. Voice indicators showed that women have more depressive tendencies than men, matching the rate of depression in Japan. The system’s voice indicators and the scores on classical psychological tests were correlated. We confirmed deteriorating mental health for users in areas affected by major earthquakes in Japan around the time of the earthquakes.

**Conclusions:**

The results suggest that although this system is insufficient for detecting depression, it may be effective for monitoring changes in mental health due to stress. The greatest feature of our system is mental health monitoring, which is most effectively accomplished by performing long-term time-series analysis of the acquired data considering the user’s life events. Such a system can improve the implementation of patient interventions by evaluating objective data along with life events.

## Introduction

The importance of mental health care is increasing globally in today’s “society of stress.” Stress negatively affects mood and health in everyday life, and accumulated stress leads to psychiatric and behavioral disorders [1]. These disorders are associated with major societal economic losses owing to lowered lifetime earnings and a decrease in labor productivity [2,3]. Early interventions for depression lead to a higher remission rate [4]; therefore, techniques to easily screen for depression and stress for the early detection of poor mental health are in demand.

Research has been conducted on screening methods for patients with mental health issues using biomarkers such as saliva [5] and blood [6]; however, these are costly as they require special measurement equipment or reagents and are invasive. Thus, noninvasive self-report psychological tests such as the General Health Questionnaire [7,8] and the Beck Depression Inventory (BDI) [9] are generally used. Although these tests are comparatively simple, the effect of reporting bias [10], in which respondents selectively underestimate or overestimate specific information either consciously or unconsciously, cannot be eliminated.

There is empirical evidence showing that mood changes are apparent in one’s facial expression and voice, and there is ongoing research estimating the depressive state and stress state using these indicators [11-14]. In addition to being noninvasive, an analysis using voice data has the particular advantage of simplicity, as it does not require specialized equipment and can be used remotely. This method also potentially solves the problems of detecting various psychiatric illnesses and eliminating the reporting bias present in self-report psychological tests. For these reasons, voice-based techniques have garnered attention in recent years.

It is desirable to continuously monitor individuals’ status to detect poor mental health as early as possible. Recently, the capabilities of mobile device platforms have rapidly improved, and smartphones can perform moderate-level arithmetic processing. Furthermore, smartphones have already become an indispensable part of our daily lives, making them the optimal tool for continuously and noninvasively monitoring elements such as users’ biological information. As such, research utilizing smartphones to detect stress and develop mental health apps for mobile terminals is actively being pursued [15-17].

In this study, we developed a system for monitoring mental health using voice data from daily phone calls, termed Mind Monitoring System (MIMOSYS) [18], by implementing a method for estimating mental health from voice data [12] through Android smartphones. It is hoped that using MIMOSYS will allow for the monitoring of daily mental health levels and the ability to circumvent conditions of poor mental health such as depression before they arise. This system was made available to the public as a prospective study in which MIMOSYS data were collected over a period of 2 years from an unspecified large sample of users who provided consent for research participation. The aim of this study was to use the data obtained to verify the effectiveness of MIMOSYS for detecting depressive states and monitoring stress-induced mental changes.

## Methods

### Ethical Considerations

This study was conducted with approval of the Research Ethics Committee of the Faculty of Medicine of the University of Tokyo (no. 10860).

### MIMOSYS

The method of voice analysis used by MIMOSYS is based on sensibility technology [19], which analyzes patterns of change in the fundamental frequency of voice data to indicate the extent of the emotions “calmness,” “anger,” “joy,” “sorrow,” and “excitement” included therein. MIMOSYS uses the emotions analyzed by sensibility technology to calculate “vitality,” the quantified mental health at the time of the call, and “mental activity,” which includes the moving average and variation in vitality measured over a 2-week period prior to the call. Mental activity is an index that is useful in monitoring changes in mental health over the mid to long term. Moving averages were taken from the previous 2 weeks because the Diagnostic and Statistical Manual of Mental Disorders-IV [20] diagnostic criteria for depression state that primary symptoms must persist for at least 2 weeks.

Vitality and mental activity were calculated using algorithms as values between 0 and 1 but were multiplied by 100 and displayed over the range of 0-100 in the app to make it easier for users to understand. Extremely low or high values were judged to represent an abnormality in mental health.

The smallest unit of speech analyzed by sensibility technology is an “utterance”; that is, continuous voice data divided by breaths and other metrics. The start of an utterance is detected as the point at which a state of silence changes to a state of speech and continues for a set period of time. The end of an utterance is detected as the point at which a state of speech changes to a state of silence for a set period of time. Distinguishing between states of speech and silence is performed by thresholding the amplitude of temporal waveforms in voice data. MIMOSYS calculates vitality for each individual utterance in voice data and outputs the average of these values as the vitality for that voice data.

### Data Collection

User consent for research participation was requested the first time MIMOSYS was used. Users who gave consent were assigned an anonymized personal ID that was saved to a dedicated server. Attribute data from a questionnaire were also saved when users registered. Attribute data included sex, age, medical history, history of present illness, and residence. All of these were self-declared. Each time a user made a call with their smartphone, MIMOSYS was automatically executed, and the analysis results were recorded consecutively on the same server. Voice data from the call were temporarily recorded on the smartphone. The analysis was performed simultaneously with the end of the call, and the voice data were immediately erased after the analysis results had been stored on the server. MIMOSYS also implemented a BDI test on the device screen every 3 months after initial use. This score was also recorded on the same server. Data were collected from July 20, 2015 to July 20, 2017.

The calls were private calls made by the users irregularly on their own smartphones. We did not impose any particular restrictions concerning the number of calls or intervals between calls.

### Participants

There were approximately 3800 total downloads of MIMOSYS during the data collection period, and consent for research participation was obtained from 2462 users. Of these, 1814 users were aged 18 and older (maximum age 81 years), for whom at least one call was analyzed by MIMOSYS. Our previous research showed that MIMOSYS does not depend on age [21], which is why this study involved users of a wide age range. MIMOSYS was downloaded by the users to their smartphones. The users had come to know about MIMOSYS through a variety of media; thus, nearly all had never met any of the researchers previously. As such, almost none of the users had attended lectures about how to use MIMOSYS in advance, and it is likely that many users had never been exposed to the BDI test conducted in the app previously. However, we assumed that they could navigate it easily as smartphone users. Anyone who agreed was qualified to participate. Users were not provided any particular reward except for being allowed to use MIMOSYS for free throughout the experimental period.

### Data Analysis

The value for vitality becomes unstable if there are few utterances in the voice data. To ensure sufficient accuracy, empirically, it is desirable that voice data include at least 6 utterances. Furthermore, we have found that to improve the accuracy of mental activities, a mental activity averaged from at least 5 vitality scores is considered empirically effective. The data meeting the above conditions were extracted from MIMOSYS user data. The data from extremely short calls (less than 10 seconds) or with unsuitable call information were excluded. Therefore, there were 183,490 (123,860, 67.50% men) entries for analysis of effective vitality and 167,610 (113,600, 67.78% men) entries for analysis of effective mental activity. However, to match users for vitality and mental activity, vitality data were excluded for users for whom effective mental activities could not be calculated. The remaining data were used as the call dataset.

Furthermore, from the above data, the first vitality score measured after the initial BDI test and the most recent mental activity score within 2 weeks of the initial BDI test were selected for each user. These data were used as the user data set and included 1015 users (651, 64.14% men).

### Analyzed Items

The following items were analyzed for the abovementioned datasets using the free statistical analysis software R version 3.4.2 [22] and G*Power version 3.1.9.2 [23].

#### MIMOSYS Rate of Continued Use

The period from the date and time each user consented to the study to the date and time of the most recent call analysis was calculated as the number of days used. Note that the number of days used was set as 0 for users who consented but did not have even one call analyzed, and for users who had a call analyzed but stopped use on the same day.

#### Normality of Vitality and Mental Activity Distributions

We examined the statistical features of the distributions of vitality and mental activity for the call dataset and for the user dataset by comparing them to the normal distribution.

#### Sex Differences in Vitality, Psychological Tests, and Vitality vs Mental Activity Comparison

The users in the user dataset were divided into those deemed healthy by the questionnaire and those deemed to have poor health, and sex differences in the vitality distribution were explored. Users were classified using the score from the initial BDI test executed by MIMOSYS and judged according to BDI evaluation criteria [24]. Based on these evaluation criteria, the foremost data after the BDI test of users with a BDI score of less than 11 were deemed to represent data from healthy individuals, whereas the foremost data after the BDI test of users with a BDI score of 11 or above were deemed to represent data from individuals with a mental health issue.

Moreover, we examined the correlation between user BDI and vitality/mental activity for the user dataset.

#### Temporal Changes in Vitality and Mental Activity Before and After a Stressful Life Event

On the nights of April 14 and 16, 2016, during the data collection period, there were two earthquakes of intensity 6 upper and 7 lower, with the epicenter in Kumamoto, Japan. According to the release by the Japanese Meteorological Agency, the earthquake on April 14 was a foreshock and that on April 16 was the main shock. Several of those affected by these earthquakes were MIMOSYS users, which allowed us to measure changes in vitality and mental activity before and after the disaster.

We then examined changes in vitality and mental activity around the time of the earthquake by area in Japan. We calculated an average of the vitality values in the 2-week periods before and after the earthquake. We also evaluated the most recent mental activity values from before the earthquake and from the 2-week period following the earthquake. The date of the earthquake was set as that of the foreshock on April 14, 2016.

## Results

### Participants

Table 1 shows the detailed participant information.

**Table 1:** Detailed information of MIMOSYS^a^ participants (N=1814).

Characteristic		Value	
Age (years), mean (SD)		39.97 (12.14)	
BDI^b^, mean (SD)		11.91 (10.37)	
Number of calls, mean (SD)		121.10 (256.58)	
Interval between calls (hours), mean (SD)		6.37 (7.73)	
Sex (men), n (%)		1135 (62.57)	
**Medical history^c^** **, n (%)**			
	Yes		855 (47.13)
	Depression		226 (12.46)
**History of present illness^c^** **, n (%)**			
	Yes		573 (31.59)
	Depression		138 (7.61)
**Residence, n (%)**			
	Hokkaido		50 (2.76)
	Tohoku		89 (4.91)
	Kanto		979 (53.97)
	Chubu		213 (11.74)
	Kansai		204 (11.25)
	Chugoku		71 (3.91)
	Shikoku		27 (1.49)
	Kyushu		151 (8.32)
	Other		30 (1.65)

^a^MIMOSYS: Mind Monitoring System.

^b^BDI: Beck Depression Inventory.

^c^“Medical history” and “history of present illness” indicate the illness(es) the user has contracted in the past and presently, respectively. The item “yes” for these categories denotes the number of users who reported a specific illness(es), and the item “depression” denotes the number of users whose specific illnesses included depression.

### MIMOSYS Rate of Continued Use

**Figure 1:**
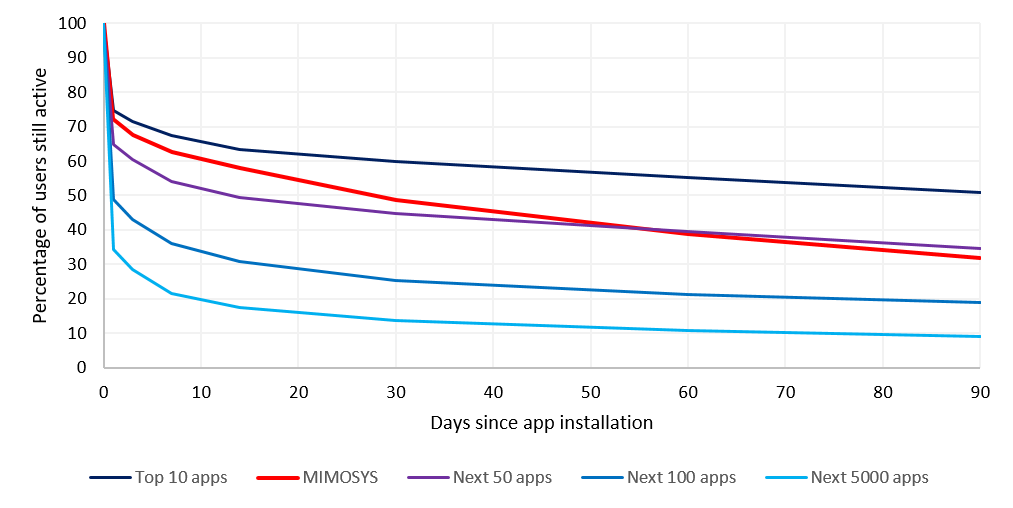
Rate of continued use curves for Mind Monitoring System (MIMOSYS) and other smartphone apps.

Figure 1 shows the rate of continued use curves for MIMOSYS and other smartphone apps. The rates of continued use for other smartphone apps referred to prior data [25]. The curves in Figure 1 for apps other than MIMOSYS show the average rate of continued use for the apps based on download rankings in Google Play. Since MIMOSYS has only been released in Japan, most MIMOSYS users live in Japan; however, a minority of people living abroad also downloaded the app in Japan.

### Normality of Vitality and Mental Activity Distributions

**Figure 2:**
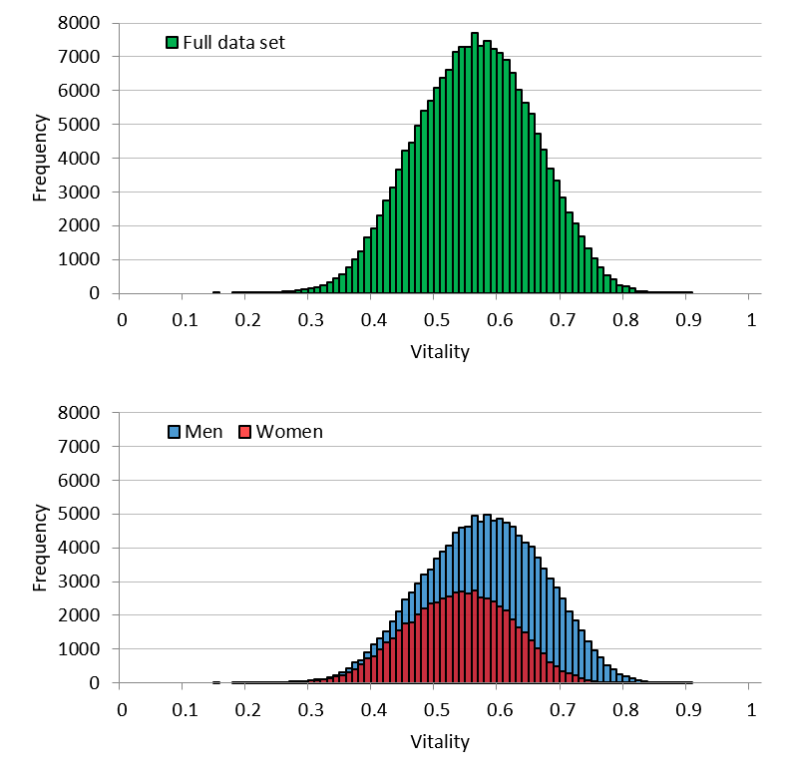
Call dataset vitality distribution for the full set and by sex.

Figure 2 shows the distribution of vitality of the call dataset. The mean of the full dataset was 0.56 (SD 0.094), whereas the mean for men and women was 0.57 (SD 0.095) and 0.53 (SD 0.084), respectively. The vitality distribution was lower for women than for men. Goodness-of-fit testing for the ideal normal distribution of the full set was significant with an effect size of 0.068 (*P*<.001) for the difference between the ideal and actual distributions [26]; therefore, the null hypothesis was rejected. Although it can be concluded that the vitality distribution does not follow a normal distribution, the effect size is extremely small. According to the effect size of evaluation criteria for the goodness-of-fit [26], there is an insignificant difference, and vitality can be regarded as having a normal distribution. Concerning the distribution for men and women separately, the goodness-of-fit effect size was 0.092 (*P*<.001) and 0.094 (*P*<.001), respectively. Thus, similar to the distribution of the full set, vitality was considered to have a normal distribution for both men and women.

**Figure 3:**
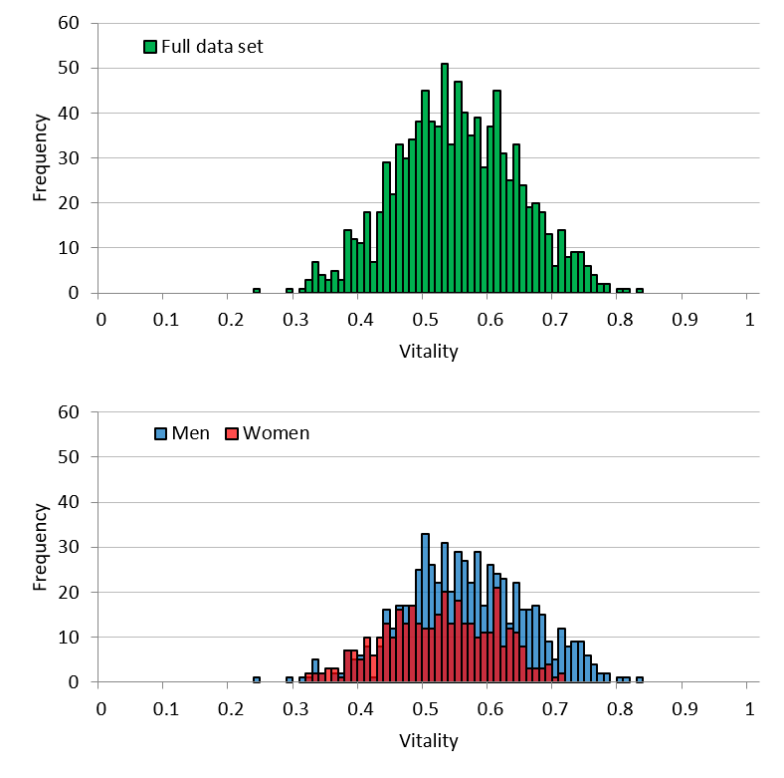
User dataset vitality distribution for the full set and by sex.

Figure 3 shows the vitality distribution for the user dataset. The mean vitality of the full dataset was 0.54 (SD 0.094), whereas the means for men and women were 0.56 (SD 0.096) and 0.52 (SD 0.085), respectively. As for the call dataset, the vitality distribution was lower for women than for men. Goodness-of-fit testing for the ideal normal distribution of the full set showed an effect size of 0.11 (*P*=.77), demonstrating that vitality follows a normal distribution. Regarding the distribution for men and women separately, the goodness-of-fit effect size was 0.15 (*P*=.68) and 0.23 (*P*=.39), respectively. Thus, similar to the distribution of the full set, it was concluded that vitality follows a normal distribution for both men and women.

**Figure 4:**
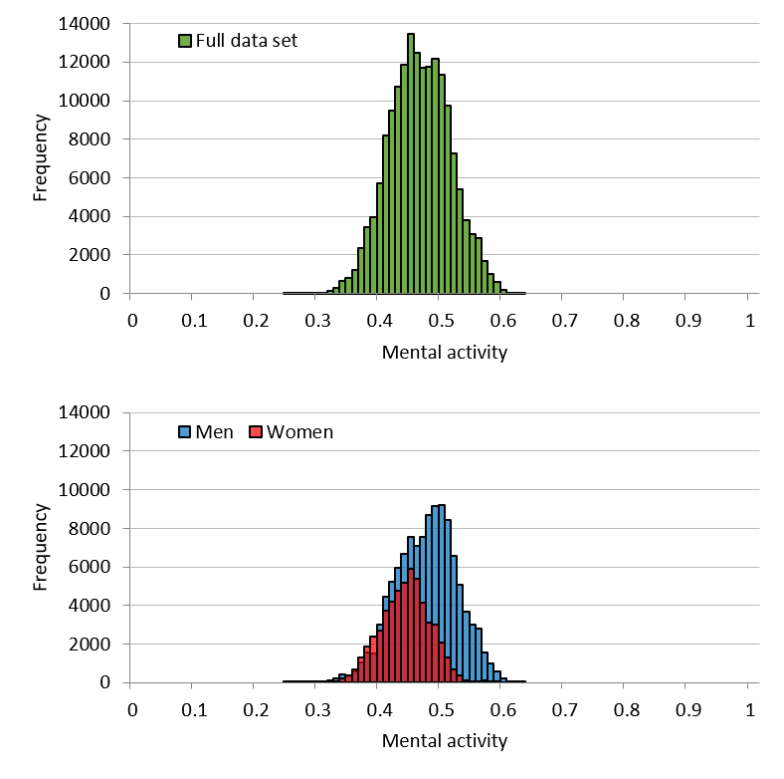
Call dataset mental activity distribution for the full set and by sex.

Figure 4 shows the mental activity distribution of the call dataset. The mean for the full dataset was 0.46 (SD 0.049), while the means for men and women were 0.47 (SD 0.050) and 0.44 (SD 0.040), respectively. As observed for vitality, the distribution for mental activity was lower for women than for men. Further, and also similar to vitality, goodness-of-fit testing for the normal distribution of the full set showed an effect size of 0.067 (*P*<.001). Therefore, mental activity was considered to follow a normal distribution. Regarding the distribution for men and women separately, the goodness-of-fit effect size was 0.085 (*P*<.001) and 0.058 (*P*<.001), respectively. Thus, mental activity was also regarded as following a normal distribution for both men and women.

**Figure 5:**
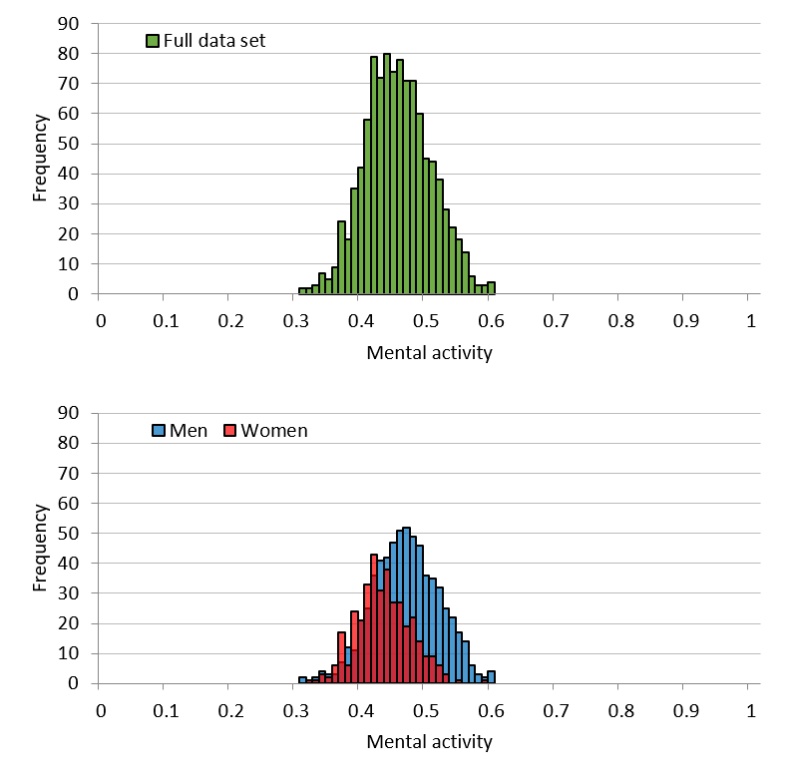
User dataset mental activity distribution for the full set and by sex.

Figure 5 shows the mental activity distribution of the user dataset. The mean for the full dataset was 0.45 (SD 0.051), while the means for men and women were 0.46 (SD 0.051) and 0.43 (SD 0.041), respectively. Similar to the call dataset, the distribution was lower for women than for men. In addition, like vitality, the effect size of the goodness-of-fit test for a normal distribution for the full set was 0.09 (*P*=.98). Therefore, we concluded that mental activity follows a normal distribution. Regarding the distribution for men and women separately, the goodness-of-fit effect size was 0.096 (*P*>.99) and 0.084 (*P*>.99), respectively. Thus, like the distribution of the full set, it was concluded that mental activity follows a normal distribution for both men and women as well.

### Sex Differences in Vitality, Psychological Tests, and Vitality vs Mental Activity Comparison

**Figure 6:**
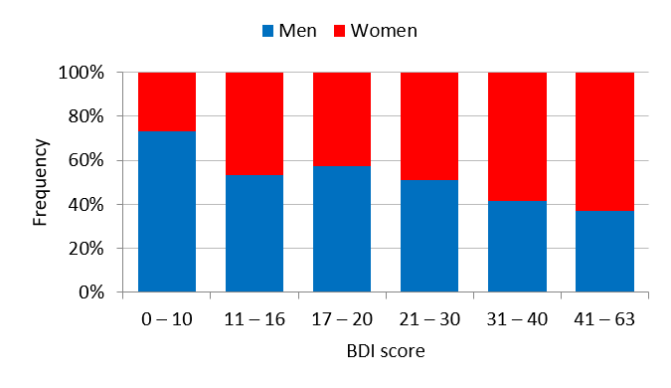
Beck Depression Inventory (BDI) score distribution by sex.

**Figure 7:**
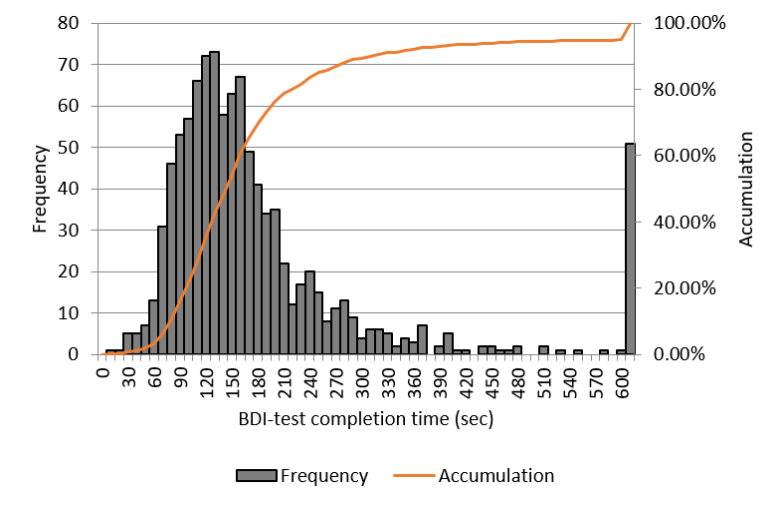
Beck Depression Inventory (BDI) test completion time distribution.

Figure 6 shows the frequency distribution of BDI scores by sex. There were more men than women with a BDI score of less than 11. In addition, the men-to-women ratio was roughly equivalent. Figure 7 shows the distribution of BDI test completion time (seconds). Approximately 10% of the participants completed the BDI test in 1.5 minutes or less. Another 10% or so of the participants took 5 minutes or more to complete the BDI test. The shortest time was 9 seconds and the longest time was about 22 hours.

**Figure 8:**
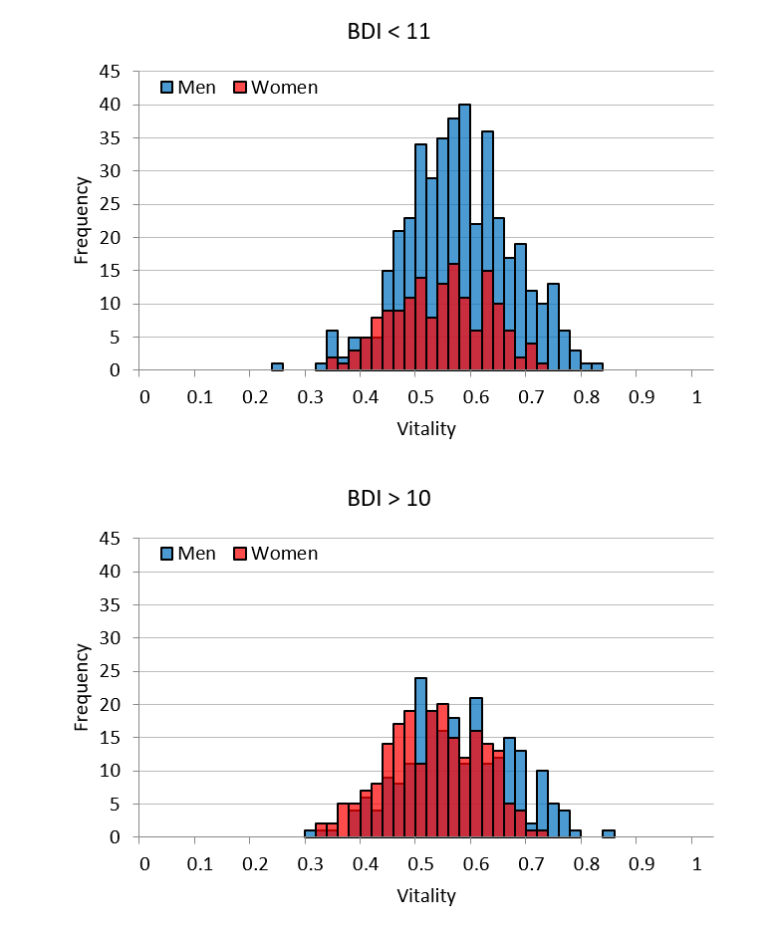
Vitality distribution by sex with Beck Depression Inventory (BDI) score evaluation criteria.

Figure 8 shows the vitality distribution by sex according to the evaluation criteria of Beck et al [24]. The *P* values shown in Table 2 represent the comparison of the mean vitality for two groups of men and women with a *t* test, and the effect size represents the size of the difference between the two groups for the *t* test [26]. Significant differences were observed in tests of the vitality for men and women in both the normal BDI score category and in the abnormal BDI score category. However, because the effect size of the difference between men and women in the normal category was considered to be small according to prior standards [26], there was no substantial difference between men and women in this category. Conversely, the effect size of the sex difference in the abnormal category was medium; thus, it was considered to indicate a substantial difference.

**Table 2:** Comparison of mean vitality between two groups of men and women according to BDI^a^ score evaluation criteria.

Category	Sex (men), n (%)	Men, mean (SD)	Women, mean (SD)	*P* value^b^	Effect size
BDI<11 (N=577)	423 (73.31)	0.56 (0.095)	0.53 (0.085)	<.001	0.34
BDI>10 (N=438)	228 (52.05)	0.56 (0.097)	0.51 (0.084)	<.001	0.48

^a^BDI: Beck Depression Inventory.

^b^Based on *t* tests.

**Figure 9:**
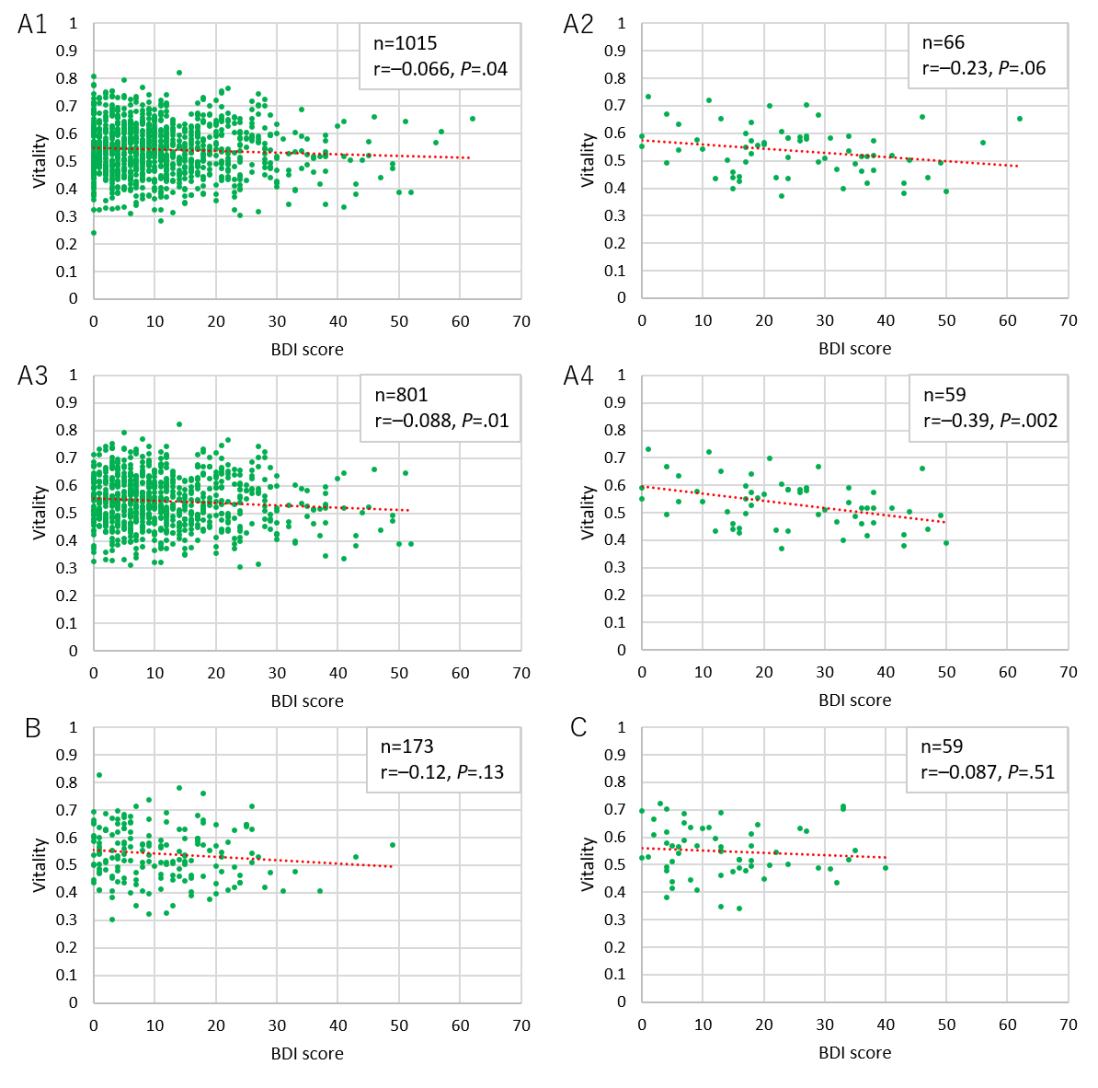
Correlation between Beck Depression Inventory (BDI) score and vitality for users.

**Figure 10:**
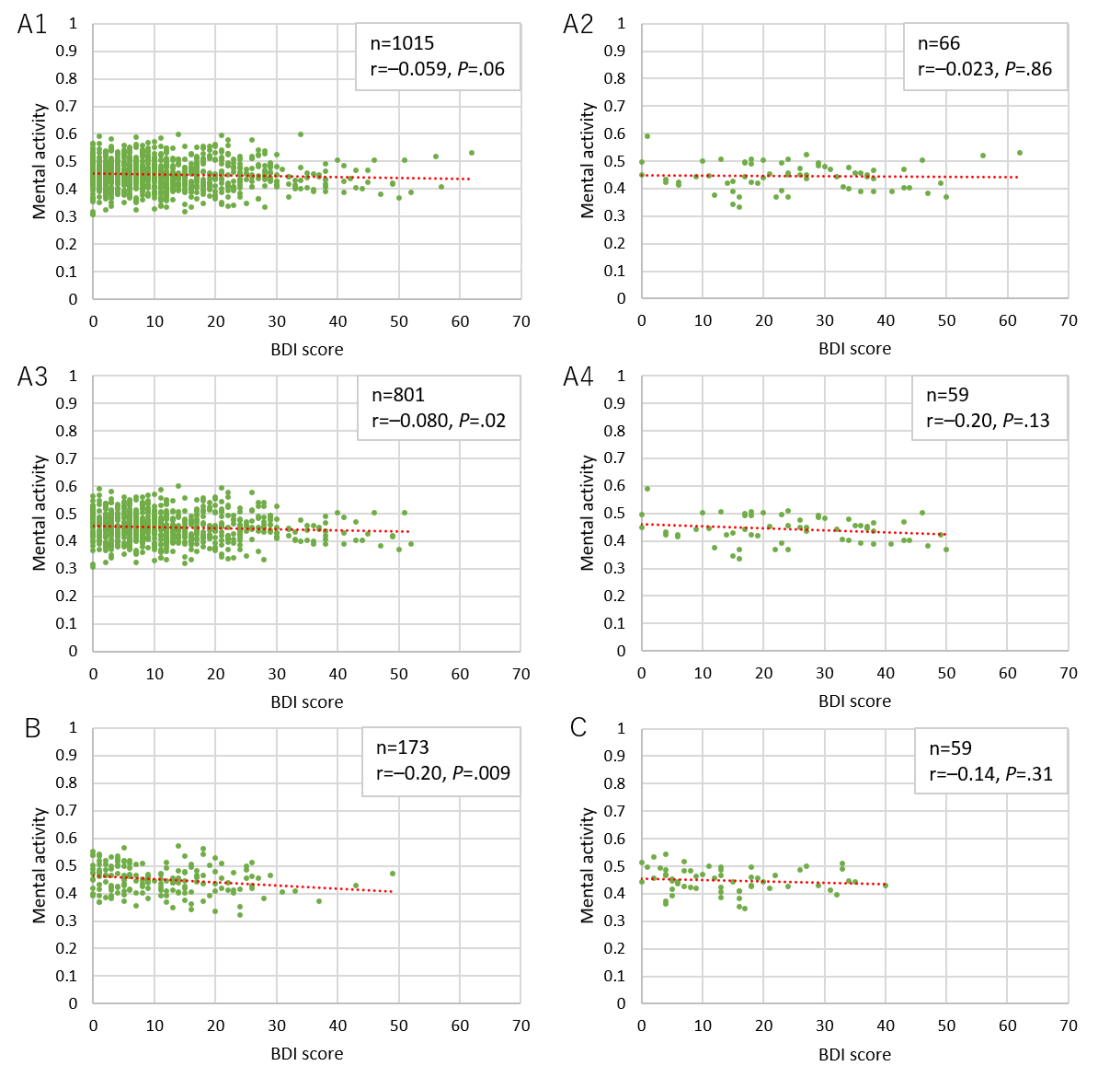
Correlation between Beck Depression Inventory (BDI) score and mental activity for users.

Figure 9 and Figure 10 show the correlations of BDI score with vitality and mental activity, respectively. Graphs A1 in both figures show the data for all users in the user dataset, graphs A2 show data for users in A1 who declared depression as a history of present illness, graphs A3 show data for users with a BDI test completion time of 80-300 seconds (all users except the top and bottom 10%), and graphs A4 show data for users in A3 who declared depression as part of the history of present illness. Moreover, graphs B show data for users from the user dataset with a completion time of 80-300 seconds in the second BDI test that was performed 3 months later. Graphs C show data for users with a completion time of 80-300 seconds in the third BDI test that was performed another 3 months later. The vitality measurements in graphs B and C were the first to be performed after the BDI test in question, whereas the mental activity measurements were the most recent values within 2 weeks of the BDI test in question. Regarding vitality, we found a significant yet weak negative correlation between the initial BDI score and vitality for users with a completion time of 80-300 seconds in the first BDI test and who declared a history depression. We did not find any sufficiently significant correlation between mental activity and the BDI score.

### Temporal Changes in Vitality and Mental Activity Before and After a Stressful Life Event

**Figure 11:**
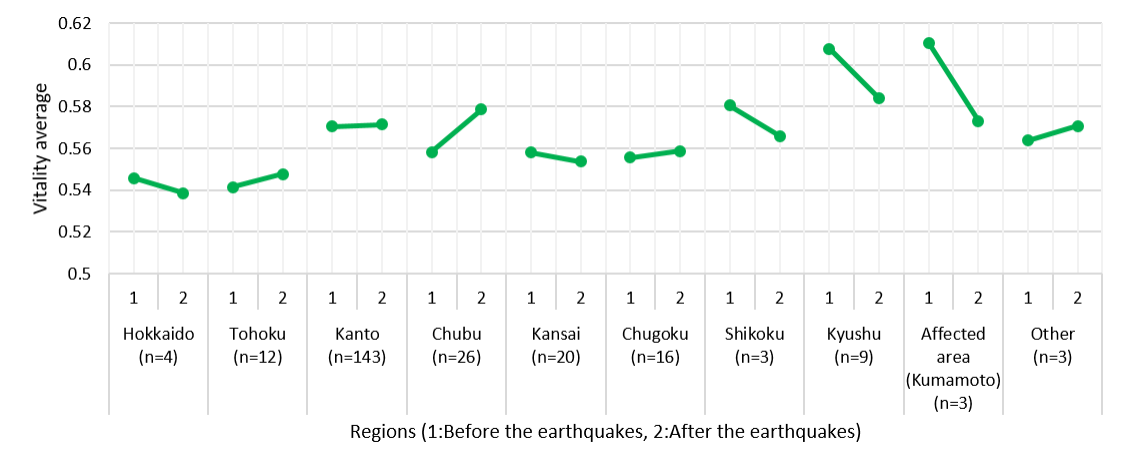
Changes in vitality before and after the Kumamoto earthquakes.

**Figure 12:**
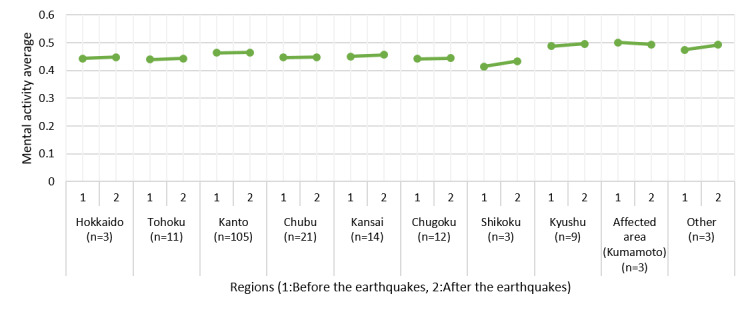
Changes in mental activity before and after the Kumamoto earthquakes.

Figure 11 shows the changes in vitality over a 2-week period before and after the earthquakes by region. Figure 12 shows the changes in the most recent mental activity before the earthquakes and within the 2 weeks after the earthquakes. After the earthquakes, vitality decreased drastically in Shikoku, Kyushu, and Kumamoto, but a slight downward trend in mental activity was observed in Kumamoto only.

**Figure 13:**
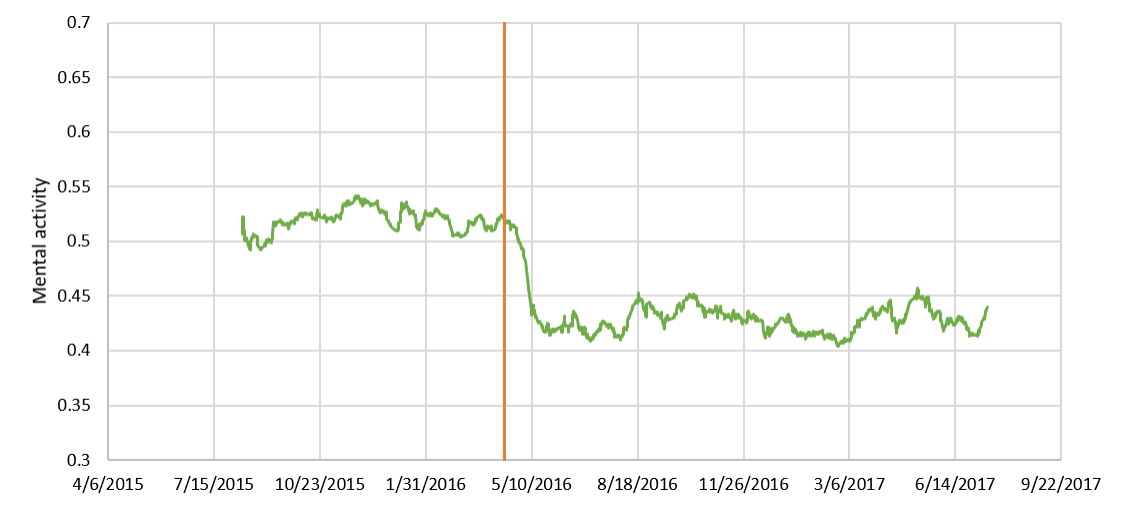
Change over time in mental activity for one victim of the Kumamoto earthquakes.

Additionally, Figure 13 shows the change over time in mental activity for one disaster victim living in Kumamoto. The vertical line in the graph represents the day and time of the foreshock. An obvious drop in mental activity can be seen before and after the earthquake. The mental activity did not return to the level before the earthquake by the end of the data collection period.

## Discussion

### Principal Findings

The rate of continued use for MIMOSYS rivaled that of the top 60 apps in Google Play’s rankings. It is believed that the primary reason contributing to long-term maintenance of a high rate of continued use is because MIMOSYS can be used effortlessly, as the app begins processing automatically when a call is made. MIMOSYS is effective at maintaining long-term use, which is a prerequisite for a monitoring system.

The distribution of vitality is considered to approximate a normal distribution because it is the average vitality for each individual utterance in the voice data. When it comes to effect size in the call dataset, it is acceptable to say that vitality follows a normal distribution. Regarding the user dataset, from the goodness-of-fit testing, we concluded that vitality follows a normal distribution. Most parametric statistical analyses assume that data for an analysis will follow a normal distribution; thus, whether the obtained data actually follow a normal distribution is vital for the statistical tests performed thereafter. From this perspective, vitality is advantageous for statistical analyses. The same can be said for mental activity. However, some distortion was detected in the distribution of mental activity. This is likely influenced by the fact that mental activity is an indicator that is largely controlled by life events unique to the user.

We have continuously observed low vitality values for people with depression in our research, whereas the vitality of healthy individuals is more widely scattered [27]. According to Kessler et al [28], approximately 20% of Japanese people in 2007 would have depression or another mental illness at some point in their lives. Since the normality of the vitality distribution showed a mean range of 0.46-0.65 for about 70% of the total, we determined that vitality values scattered within this range indicate good mental health, which is in line with the report of Kessler et al [28].

Women tended to have lower vitality and mental activity than men. There are two probable reasons for this. The first is a problem with the algorithm. Women are generally more expressive of their emotions than men [29]. The differing trends in emotional expression between men and women were accounted for in the development of MIMOSYS through the proposal of an algorithm designed to eliminate sex differences [27]. However, the difference may have surfaced with the increase in the size of the dataset analyzed because the number of data entries from teachers used in the development of the algorithm was insufficient.

The second reason is the underlying sex difference in the rate of depression, which is higher in women than men [30]. In the distribution of BDI scores, there were overwhelmingly fewer women than men in the normal category, and the more severe category also had slightly more women than men. It is possible that MIMOSYS is detecting this difference. To investigate this, data were classified based on the evaluation criteria for BDI score and sex differences in vitality explored for each category. In the normal category, from the viewpoint of effect size, there was no substantial sex difference considered to exist. Conversely, in the abnormal category, from the viewpoint of effect size, there was a substantial sex difference determined. In other words, the MIMOSYS algorithm was functioning correctly in the normal group, whereas the abnormal group may be reflecting differences in the rate of depression. Nonetheless, the difference in effect size between the normal and abnormal groups was not overly large, and this point requires additional investigation.

We could not find any sufficient correlation between BDI score and vitality/mental activity in this study. A possible reason for this is that it typically takes 2-3 minutes to complete the BDI test if conducted normally; however, we observed a wide spread of completion times that might not have yielded reliable scores. As such, we conducted the analysis after excluding the top and bottom 10% of completion times as they were considered to have low reliability; however, this did not lead to any considerable changes. The reason for this is unknown. We observed the same tendencies for the BDI scores in the second and third tests performed at 3-month intervals. We believe that users with depression responded to the test relatively seriously and found a significant but weak correlation between vitality and initial BDI score; yet, these data cannot be considered sufficient. As discussed below, the data from the Kumamoto earthquakes clearly traced changes in mental state. This suggests the possibility that BDI testing through smartphones, the gap between reality and self-reported conditions because of reporting bias, and the scattering of voice evaluation mutually influenced the results. As such, it is possible that this system is more suitable for monitoring rather than for screening if we take the BDI test as the standard. Furthermore, the reason we did not refer to only users with depression in the second and third tests is that we could not find a sufficient number of users, which undermined the reliability of the statistical analysis.

The reason we could not find any correlation between BDI score and mental activity for users with depression is likely the same as that for vitality. Moreover, since we did not have any vitality data collected prior to BDI score measurement, it is possible that the actual tendency for the correlation with the initial BDI score was not reflected in the mental activity. In fact, we did find an extremely weak yet significant correlation with the second BDI score. Nevertheless, any further investigation would be limited since we suspect a major influence from the reliability of the user data.

We previously reported a correlation between BDI score and vitality/mental activity [31,32]. We also reported a correlation between vitality and the Hamilton Depression Rating Scale [33] for individuals with depression [34]. These results were obtained from participants who were recruited appropriately, which supports that this method exhibits adequate performance under specified conditions. Although it may also be possible to obtain a favorable result from these data by extracting information from users whose day-to-day background is clear, it is difficult to know a user’s detailed state from their attributes; thus, reexamining the study design remains a future research task. However, the design used in this study has shown us that it is difficult to obtain our intended result.

As the BDI test is intended for clinical applications, it would have been more appropriate to use Patient Health Questionnaire 9 [35] and Center for Epidemiologic Studies Depression Scale [36] for this study. However, it was not possible to additionally administer these tests to the participants; therefore, they should be utilized in future studies.

As the primary purpose of MIMOSYS is mental health monitoring, analyzing changes in vitality and mental activity in relation to the user’s life events is desired. We observed a deterioration of mental health among users in the areas affected by the Kumamoto earthquakes around the time of the disaster. This was thought to reflect the heightened stress caused by the earthquake. It is easiest to understand this finding by considering the results of one disaster victim presented as an example. This user’s mental activity was good for some time after starting to use MIMOSYS; however, there was a steep drop immediately after a certain point, following which mental activity did not recover by the time the user stopped using MIMOSYS. The Kumamoto earthquakes occurred around the time of the marked change in mental activity, and it is hypothesized that the cause of the drop in mental activity was stress from the earthquakes. By observing changes in vitality and mental activity along with life events over the mid to long term in this manner, we can not only detect poor mental health in the early stage but can also hypothesize the cause of that state, leading to a solution at the root of the problem.

From this perspective, although MIMOSYS is not well-suited for taking measurements upon every screening, the results suggested that it could be an effective mental health monitoring system. A separate study in which we compared changes in vitality and mental activity in the affected area and other regions at the time of the Kumamoto earthquakes [37] showed that vitality and mental activity increased in a region in which a similar earthquake had occurred in the past (Tohoku) because of excitement from flashbacks to memories of that time (trends similar to those can be observed in Figures 11 and 12 as well). This result may reflect detecting symptoms of posttraumatic stress disorder such as hypervigilance or irritability, and indicates that MIMOSYS may be useful in detecting posttraumatic stress disorder. Nevertheless, we did not conduct any examination using the Impact of Event Scale-Revised [38] or the Post-traumatic Diagnostic Scale [39]; thus, such comparisons are a task for future studies.

This discussion assumes the legitimacy of users’ attribute data; however, because these data were reported by users for the current study, it is difficult to verify the reliability. Therefore, it is possible that accuracy was lost when categorizing the data. Moreover, since the voice analysis was conducted using smartphones privately owned by the users, we could not exclude dependence on the model. This is a limitation of the current study and a challenge for future research. Data collection via smartphone is simple and allows for the accumulation of large-scale data; however, we encountered the problem of necessarily rejecting the null hypothesis in statistical hypothesis testing because of a too-large sample size. In the future, it will be necessary to include not only analyses based on frequentism but also Bayesian statistics.

### Conclusions

In this prospective study, a system for monitoring mental health based on voice data from smartphone calls (MIMOSYS) was opened to the public, data were collected over a period of 2 years from a large unspecified sample of users, and the characteristics and potential of MIMOSYS were examined from various perspectives to verify the effectiveness of the system.

MIMOSYS was found to be effective concerning the rate of continued use. The greatest feature of MIMOSYS is mental health monitoring, and this is done most effectively by performing long-term time-series analysis of mental activity considering users’ life events. Fluctuation in mental activity because of stress from life events was observed. It is likely that patient interventions will be facilitated by evaluating objective data such as those collected by MIMOSYS together with life events. However, it is difficult to collect information on users’ personal life events with this system alone. Hereafter, we wish to investigate a method of data collection that accounts for this and leads to further verification of MIMOSYS.

## Abbreviations

BDI: Beck Depression Inventory
MIMOSYS: Mind Monitoring System
